# Team-based primary care without collaboration: a call for academic partnerships in relational transformation

**DOI:** 10.3389/fmed.2026.1803192

**Published:** 2026-03-25

**Authors:** Andrew McLellan, Asiya Odugleh-Kolev, Vanessa Wright, Joanna Binch

**Affiliations:** 1 Master of Nursing – Nurse Practitioner Program, Faculty of Health Disciplines, Athabasca University, Athabasca, AB, Canada; 2 Department of Integrated Health Services (IHS), World Health Organization, Geneva, Switzerland; 3 Lawrence Bloomberg Faculty of Nursing, University of Toronto, Toronto, ON, Canada

**Keywords:** academic-practice partnerships, interprofessional collaboration, primary healthcare, relational practice, team-based primary care

## 1 Introduction

Team-based primary care is promoted globally as essential to strengthening primary healthcare (PHC) and advancing universal health coverage (UHC). Internationally, team-based primary care has expanded beyond physician-exclusive models to respond to increasing primary care demands (1). Within PHC reform agendas, team-based models are promoted as a way to enhance coordination, continuity, and overall quality of care by bringing together diverse health professionals to address complex population health needs (2). However, evidence from diverse primary care settings reveals a troubling pattern: professionals who work alongside each other in team structures continue to practice in parallel, with limited shared decision-making, fragmented care planning, and poorly integrated workflows (3). There is an underlying assumption that the skills to successfully work as part of a team develop organically, rather than recognizing interprofessional collaboration is a learned skill. The presence of teams does not reliably produce collaboration.

Collaborative practice is inherently interprofessional and occurs when multiple health workers from different professional backgrounds work with patients, caregivers, families and communities to deliver the highest quality of care (4). This paper argues that the persistent gap between team-based organization and collaborative practice reveals a deeper conceptual and pedagogical failure rooted in mechanistic thinking. It cannot be explained by insufficient structural reform alone. When health systems are conceptualized like clockwork mechanisms, where problems can be broken down, analyzed, and solved through structural redesign, the solution to collaboration appears straightforward: co-locate professionals, diversify roles, create coordination mechanisms, and collaboration will emerge (5). This logic has dominated healthcare organization and workforce design for decades.

## 2 Complexity, emergence, and relational practice

The shift toward viewing health systems through a complexity lens has accelerated, largely in response to the shortcomings of traditional, reductionist, and linear management approaches (6). Health systems are now understood as complex adaptive systems characterized by fuzzy boundaries, dynamic interactions, unpredictability, and emergent properties that cannot be reduced to their component parts (7, 8). In such systems, collaboration is not an automatic output of structural design. Rather, it is an emergent phenomenon that arises from the quality of relationships and interactions among professionals, patients and families, and communities. This is where relational practices become central. It was previously thought that human skill, capital, motivation, and commitment were the dominant forces in high performance teams. It has since become clear, however, that intra and extra-team relationships are pivotal for team functioning (9) The WHO-WISH Integrated Change Framework (ICF) conceptualizes relationality as an ongoing process of relationship-building grounded in trust, shared purpose, and co-production, operating simultaneously across individual, team, organizational, and system levels (10). Relational practice further describes the value and development of relationships or connections with others, and is a key concept across health and education systems (11). Relational practices include shared sense-making, dialogic communication, collective reflection, negotiated decision-making, and navigating power and difference. These extend far beyond interpersonal skills. Rather than functioning solely at the individual level, these practices operate as systemic mechanisms through which collaborative capacity can be built and sustained.

Without relational practice, the persistent gap between team-based structures and collaborative practice becomes intelligible. Structural reforms create potential spaces for collaboration; however, without explicit attention to relationship building across multiple system levels, these spaces remain unrealized. Team-based primary care, as it is currently conceptualized, risks reproducing hierarchical and siloed models under the appearance of collaboration, fundamentally limiting the transformative intent of PHC reform.

## 3 Evidence of relational transformation

Evidence from the WHO *Community Engagement Research Initiative* in the Western Pacific Region demonstrates that relational practices are a key mechanism through which collaboration is enabled in primary healthcare (12). Drawing on multi-country case studies, including Lao People's Democratic Republic, Cambodia, and Malaysia, the evaluation shows that intentional investment in trust-building, dialogue, shared problem-solving, and feedback between communities, frontline providers, and local authorities strengthened coordination, improved service responsiveness, while supporting more integrated PHC delivery. Across these settings, collaborative capacity emerged not from structural arrangements alone, but from deliberate attention to how relationships were built and sustained in practice.

## 4 The pedagogical challenge

Health professional education remains largely structured around disciplinary silos, with interprofessional education treated as an add-on rather than a foundation. When relational competencies are not explicitly taught through appropriate pedagogies before professionals enter practice, graduates arrive at team-based primary care settings underprepared for the relational work that collaboration requires. In-service training attempts to remedy this but faces the challenge of shifting established professional identities, power dynamics, and entrenched practice patterns.

The pedagogical failure is thus systemic, pre-service education produces professionals who are socialized into disciplinary boundaries and practice environments, and rewards individual technical competence over relational capacity. Moreover, organizational structures and funding mechanisms reinforce parallel rather than collaborative work; and continuing professional development often lacks the sustained, practice-based approach necessary for relational transformation.

The importance of relational practice is reflected in the WHO Framework for Action on Interprofessional Education and Collaborative Practice. The Framework defines collaborative practice as health workers from different professional backgrounds working together with patients, families, and communities to deliver high-quality care across settings, and explicitly positions interprofessional education as foundational to developing this capacity (4). This framing underscores that collaboration must be intentionally cultivated through education rather than presumed to arise through proximity or policy. Similarly, the Interprofessional Education Collaborative (IPEC) Core Competencies for Interprofessional Collaborative Practice (13) emphasize relational competencies, including relationship-building, communication, shared leadership, conflict management, reflection, and team reasoning, as central to collaborative practice. These are competencies that can be taught, practiced, and assessed.

Recent WHO work on relationality extends this foundation by articulating how relational capacities can be built and sustained across health systems. The WISH–WHO Integrated Change Framework conceptualizes relationality as an ongoing process of relationship-building grounded in trust, shared purpose, and co-production. It also specifies how relational practices operate across individual, team, organizational, and system levels (10). Importantly, this work moves beyond defining collaborative competencies to demonstrating how relational capacities can be embedded through education, leadership, organizational routines, and evaluation. In doing so, it positions relational practice not as an abstract value, but as a mechanism through which collaboration is learned, enacted, and sustained in support of PHC reform.

## 5 A call to action: priorities for academic–practice partnerships

Addressing these challenges requires academic institutions and health systems to form partnerships that fundamentally transform how collaboration is understood, taught, and sustained. We therefore propose four interconnected priorities to guide academic-practice partnerships.

### 5.1 Transformation of pre-service health professions education

Health professional curricula must be redesigned to position relational competencies as essential, rather than supplementary. This requires interprofessional education to begin in the earliest stages of professional formation, before disciplinary boundaries and professional identities solidify around individual autonomy as the dominant marker of competence. Academic partnerships across institutions and faculties need to co-develop and test pedagogies that create relational learning environments where students experience collaborative practice through extended clinical opportunities in team-based settings. This includes structured observation and assessment of relational practices, as well as simulation and role-play that address power dynamics and conflict. Embedded reflective practices that build capacity for uncertainty and continuous learning help prepare health professions for real world practice settings. Continued investment is also needed to refine and expand assessment approaches that measure the impact of interprofessional education (14).

### 5.2 Development of faculty and practice educators

Educators in both academic and practice settings must themselves develop relational competencies and pedagogical approaches appropriate to complexity. The discourse of complexity requires continued exploration within and across these settings. Academic- practice partnerships across health and social care arenas can facilitate this dialogue and ground it in practice. Within academic settings, academic–practice partnerships should establish faculty development programs that model effective collaborative practice, facilitate collective reflection and learning, assess relational competencies in authentic contexts, and foster psychologically safe learning environments. Attention needs to be given to navigating power differentials within interprofessional teaching teams, challenging traditional healthcare hierarchies, and co-creating objectives and partnerships.

### 5.3 Paradigm shift in research and evaluation of primary care

Academic-practice partnerships must embrace complexity-informed research methodologies that study collaboration as it unfolds in complex adaptive systems. This requires moving beyond randomized controlled trials that seek to demonstrate causality in closed systems and toward adaptive, participatory research designs that explore how relational practices emerge, spread, and sustain across contexts (5, 15). Research should examine collaboration not as a dependent variable produced by structural inputs but as an emergent property of multi-level relational dynamics, using complexity-informed action research and systems mapping. Given the systems-oriented nature of this inquiry, the study of health services must engage all people and groups who interface with these environments, including professionals, educators, researchers and communities, across academic faculties and practice settings.

### 5.4 Integration of relational capacity-strengthening in health systems

An important role for academic partnerships is to support for health systems in embedding relational practices across organizational levels. This includes advancing leadership development that emphasizes adaptive and transformative approaches, establishing relational competency benchmarks for hiring and performance evaluation, and creating structured mechanisms for collective reflection such as action learning sets, after-action reviews, and appreciative inquiry. These partnerships should also support the mapping and assessment of community-member relationships in priority care networks, and the integration of relational indicators in performance monitoring and decision-making processes. Ultimately, the intentional merging of pedagogy, academia, and practice is essential to build the health system capacity required to fully realize relational practice.

## 6 Conclusion

The widespread failure of team-based primary care to produce collaboration is not a problem requiring more sophisticated structural design but a fundamental challenge demanding pedagogical and systemic transformation. Health systems are complex adaptive systems where collaboration emerges from intentionally cultivated relational practices operating across individual, team, organizational, and system levels.

Academic institutions cannot remain bystanders to this challenge. While academic institutions play a pivotal role in shaping professional formation, responsibility for relational transformation extends beyond the academy. Health systems, professional associations, and practice environments also have critical roles in cultivating, reinforcing, and sustaining relational capacity through in-service education, leadership development, and organizational design. As architects of professional identity formation and generators of implementation science, academic institutions must partner with health systems to lead the shift from mechanistic thinking to complexity-informed, relational approaches. This requires transdisciplinary research collaborations, transformation of curricula and pedagogies, faculty development, and sustained engagement with practice environments.

Education, curriculum, and pedagogy therefore sit at the center of the challenge facing team-based primary care. When relational competencies are not explicitly taught, practiced, and assessed through appropriate pedagogical approaches, graduates enter team-based primary care settings underprepared for the relational work that collaboration requires (10–13). In such contexts, team-based care risks becoming merely a structural change, lacking the relational foundation needed for interprofessional collaboration to fully advance primary healthcare reform. Longitudinal investment across pre-service, graduate and continuing professional development is required within academic partnerships to achieve this goal for the health professions. This paper reframes collaboration in team-based primary care as a conceptual and pedagogical problem and argues that relational practices, intentionally cultivated across individual, team, and organizational levels, are the essential mechanism for transforming team-based structures into genuinely collaborative primary healthcare.

Without relational transformation, team-based primary care will continue to reproduce fragmented and hierarchical models under new labels, failing to realize the vision of PHC as comprehensive, coordinated, people-centered care.
